# A Multidisciplinary Approach in the Management of a Paediatric Posterior Fossa Ischaemic Stroke: A Case Report

**DOI:** 10.7759/cureus.6418

**Published:** 2019-12-19

**Authors:** Orazio Buonomo, Antonio Armando Caragliano, Agostino Tessitore, Antonio Pitrone, Sergio Lucio Vinci

**Affiliations:** 1 Neuroradiology Unit, Biomedical Sciences and Morphologic and Functional Images, University of Messina, Messina, ITA

**Keywords:** posterior fossa ischaemic stroke, endovascular treatment, occipital craniectomy, multidisciplinary approach in stroke care, paediatric ischaemic stroke, vertebral artery dissection, basilar artery occlusion

## Abstract

Posterior circulation acute ischaemic stroke in childhood is a rare but life-threatening disease. We describe a paediatric case of a 17-year-old Indian boy who was admitted to our centre for headache, nausea, vomiting, asthenia, and fever for two days. Computed tomography angiography (CTA), magnetic resonance angiography (MRA) and digital subtraction angiography (DSA) were performed, showing a thrombotic occlusion of the basilar artery due to focal dissection into the proximal third of the left vertebral artery. In a multidisciplinary fashion, we decided to perform a direct aspiration first pass technique (ADAPT), which led to the complete recanalization of either the left vertebral artery or the basilar artery. Twenty-four hours later, despite the anti-edemigenic medical therapy, a preventive occipital craniectomy was performed because of the presence of cerebral oedema to avoid the possible worsening of the patient and compression on the brainstem.

Our experience emphasizes the importance of a multidisciplinary and preventive approach in the management of a paediatric posterior fossa ischaemic stroke.

## Introduction

Currently, paediatric stroke is a challenging disease that makes the approach of a physician highly complex, especially because uniform and accepted guidelines do not yet exist. According to Felling et al., paediatric ischaemic stroke has an estimated adjusted incidence of 1.6 x 100,000 per year [1], which is lower than the incidence of stroke in adults, and the overall lifetime risk of acute ischaemic stroke is, on average, 18.3% in Western Europe [2]. In adults, posterior fossa strokes occur four times less frequently than anterior circulation strokes and are generally linked to atherosclerotic disease [3]. In children, posterior fossa strokes were linked (45%) to vertebral artery dissection (V2-V3 traits), mainly trauma-related dissection [4-5]. Posterior fossa ischaemic stroke has an important sex-related incidence (male/female: 9/1). The most common symptoms (headache, neck pain, nausea, and vertigo) do not always lead to an immediate diagnosis of the pathology [4]. As a result, a long time often elapses between the onset of symptoms and a definitive diagnosis, which can lead to unfavourable long-term clinical outcomes and even death.

In these cases, it is essential to take care of the patient in a high-volume centre with many diagnostic tools (computed tomography (CT), magnetic resonance imaging (MRI), and digital subtraction angiography (DSA)) and multidisciplinary participation by several specialists (neurologists, neuroradiologists, neuroanaesthetists, and neurosurgeons) who must work together to improve patient management [6-7].

## Case presentation

We present the case of a 17-year-old Indian boy who was transferred from a peripheral centre to our hospital due to the persistence and worsening of his clinical status. The symptoms started two days before the transfer and included headache, nausea, and vomiting. The cause of the symptoms was not determined at the peripheral centre; hence, telephone consultations occurred. In the afternoon of the third day, the young boy was transferred directly to the stroke unit of our hospital.

Upon his arrival, asthenia and fever were also revealed. The neurological physical examination showed the presence of drowsiness, left hemiparesis, sixth cranial nerve palsy bilaterally, and positive responses bilaterally to Babinski reflex tests. The examination also showed the absence of the corneal reflex bilaterally and small reactions of the isochoric pupils to light.

Before he was transferred to our hospital, a brain CT scan of the patient was taken which showed a hypodense area in the right cerebellar hemisphere with other hazy hypodensities in the left cerebellar hemisphere. Computed tomography angiography (CTA) of the brain documented an occlusion at the top of the basilar artery and the absence of the V4 segment of the left vertebral artery.

**Figure 1:**
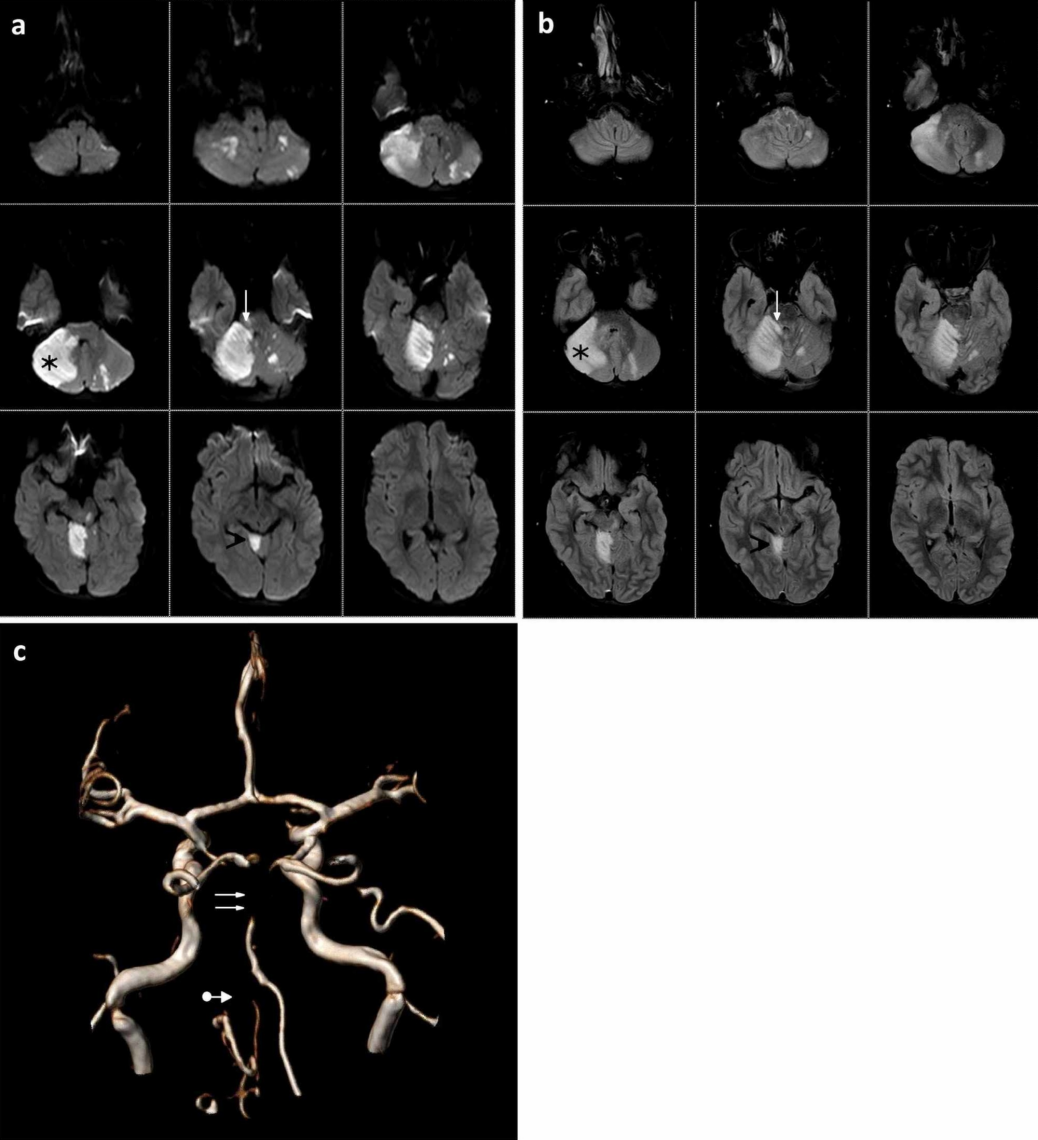
Admission magnetic resonance imaging (MRI) An MRI scan taken upon the patient’s arrival that demonstrates: a) a hyperintense hyperacute ischaemic area in the right cerebellar hemisphere (black star) and in the right-sided vermis (black arrowhead) with partial involvement of the right middle cerebellar peduncle and the upper cerebellar peduncle (white arrow) in diffusion-weighted imaging (DWI) sequence; b) a hyperintense acute ischaemic area in the right cerebellar hemisphere (black star) and in the right-sided vermis (black arrowhead) with partial involvement of the right middle cerebellar peduncle and the upper cerebellar peduncle (white arrow) in fluid-attenuated inversion recovery (FLAIR) sequence; c) magnetic resonance angiography (MRA) shows the absence of flow in the middle-distal tract of the basilar artery (double white arrows) with a reduction similar to that of both vertebral arteries (white arrow with dot)

Once he arrived, the patient was subjected to a brain MRI scan, which revealed a markedly hyperintense lesioned area in the right cerebellar hemisphere with partial involvement of the right middle cerebellar peduncle and the upper cerebellar peduncle in both diffusion-weighted imaging (DWI) and fluid-attenuated inversion recovery (FLAIR) sequences with a consensual hypointensity in the apparent diffusion coefficient (ADC) map. In addition, other circumscribed DWI hyperintensities of the left cerebellar hemisphere and in the brainstem in the paramedian left pons were detected (Figure 1a-b).

These lesions were correlated with ischaemic events in the acute phase. An Alberta Stroke Program Early CT Score for posterior circulation (pc-ASPECTS) of 6-5 points was calculated according to the recently published guidelines [8]. Susceptibility weighted imaging (SWI) sequences documented small foci of blooming in ischaemic areas related to blood infarction without a space-occupying effect (HI-1) [9]. Magnetic resonance angiography (MRA) showed the absence of flow in the middle distal tract of the basilar artery with a reduction similar to that of both vertebral arteries (Figure 1c).

**Figure 2:**
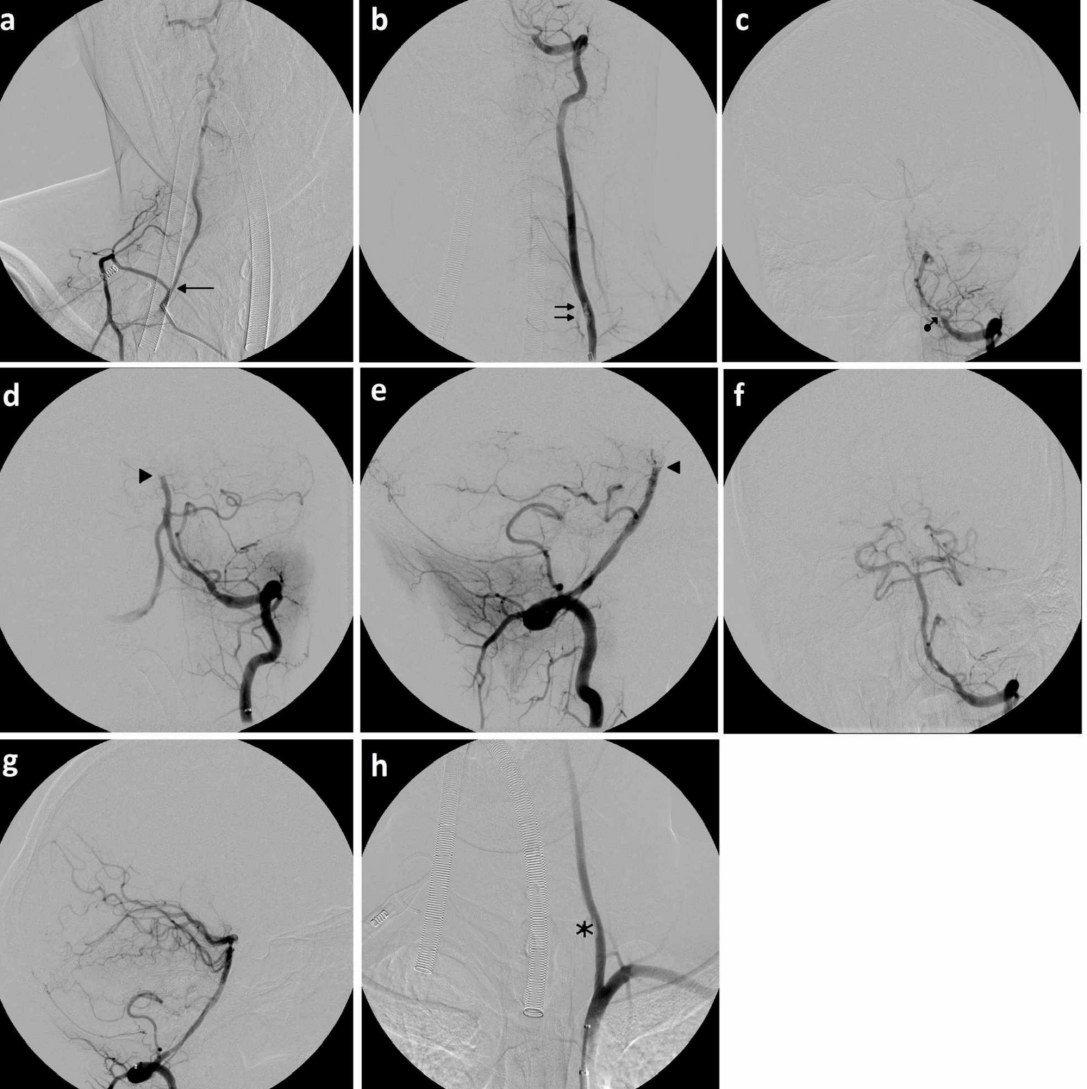
Endovascular treatment The digital substraction angiogram (DSA) images demonstrate the various phases of treatment: a) an anteroposterior (AP) angiogram of the right vertebral artery shows the common origin (black arrow) of the artery with a costocervical trunk; b) AP angiograms of the left vertebral artery in the neck and intracranial areas show a focal length dissection of the proximal V2 trait (double black arrows) and (c) thrombotic occlusion (black arrow with dot) immediately adjacent to the origin of the posterior inferior cerebellar artery (PICA); d-e) AP and latero-lateral (LL) angiograms of the left vertebral artery after the first pass with the catalyst aspiration catheter show the basilar artery occluded at the apex (black arrowheads); f-g) AP and LL angiograms of the left vertebral artery after the second pass with the catalyst aspiration catheter demonstrate normal posterior circulation (modified thrombolysis in cerebral infarction (mTICI 3)); h) AP angiogram of the left vertebral artery in the neck shows the stability of the focal length dissection of the proximal V2 trait (black star) with a regular and orthograde flow.

Considering the clinical examination and the imaging results, the patient was taken to the angiography room to perform a cerebral DSA. The angiographic procedure was performed under general anaesthesia with an 8-French right transfemoral arterial access. Angiograms showed the thrombotic occlusion of the left vertebral artery immediately near the origin of the ipsilateral posteroinferior cerebellar artery (PICA) and the presence of thrombotic material (probably due to focal dissection) into the proximal third of the vertebral artery itself. Furthermore, through arteriography of the right vertebral artery, occlusion was noted at the level of the basilar artery apex (Figure 2a). The perfusion of both posterior cerebral arteries was guaranteed by anterior circulation through both posterior communicating arteries.

Based on the angiography results, it was decided, in a multidisciplinary fashion, to implement a direct aspiration first pass technique (ADAPT) attempt with an aspiration catheter (AXS Catalyst 6F) with an inner diameter of 0.60 inches (Stryker Neurovascular, Fremont, CA, USA), which led to the complete recanalization of either the left vertebral or the basilar artery (Figure 2b-g). The angiographic checks at the end of the procedure revealed the presence of irregularities in the left vertebral artery medial wall referring to a dissection intimal flap (Figure 2h).

**Figure 3:**
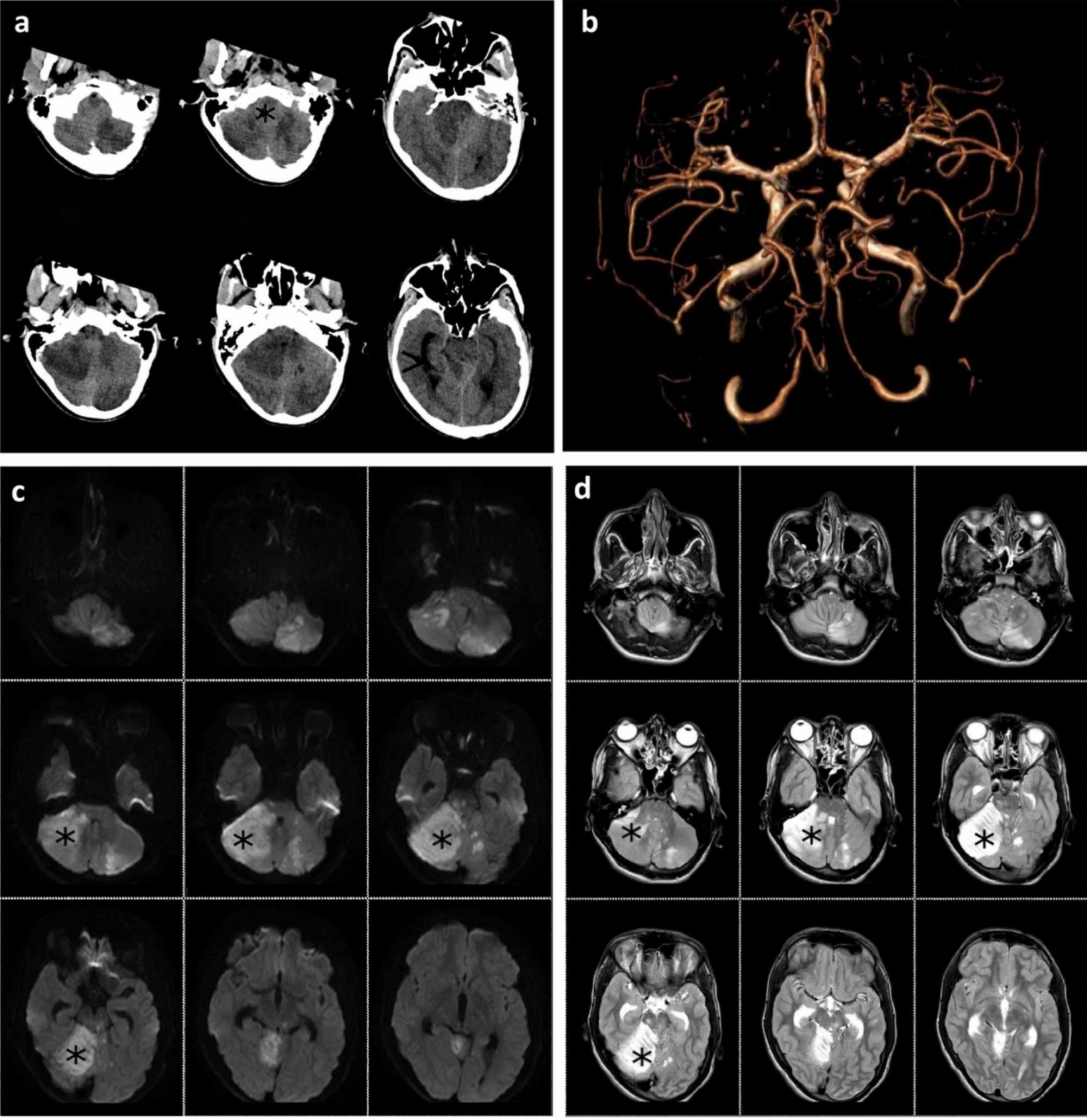
Computed tomography (CT) and magnetic resonance imaging (MRI) examination after endovascular treatment a) Six hours after treatment, a CT scan showed the previously diagnosed ischaemic outbreaks in addition to initial dilatation of the supratentorial ventricular system (black arrowhead) and a reduction of the Silvio aqueduct and the fourth ventricle (black star), due to cerebral cytotoxic oedema; b) 24 hours after the endovascular procedure, magnetic resonance angiography (MRA) showed typical posterior circulation; c) in diffusion-weighted imaging (DWI) sequence and d) in fluid-attenuated inversion recovery (FLAIR) sequence, the ischaemic lesion (black stars) is evident with the same findings as those seen in the last CT scan. Immediately after MRI, a preventive occipital craniectomy was performed.

After the endovascular treatment, the patient was transferred to the intensive care unit (ICU) under the continuous monitoring of vital parameters. Six hours after treatment, a brain CT scan documented the previously diagnosed lesion outbreaks, but it also showed the initial dilatation of the supratentorial ventricular system with the reduction of the Silvio aqueduct and the fourth ventricle due to cerebral cytotoxic oedema (Figure 3a).

Therefore, anti-edemigenic medical therapy was initiated. The patient received a 3.75 ml/kg intravenous infusion of 20% mannitol. Twenty-four hours after the endovascular procedure, a sedation window was used to assess the patient's clinical condition. The assessment revealed improvements in the neurological symptoms, including the spontaneous opening of the eyes, spontaneous movements of the four limbs with residual deficits in the distal portion of the left upper limb, present and symmetrical osteotendinous reflexes, and a positive response to the Babinski sign on the left side.

After a neurosurgical consultation, a preventive occipital craniectomy was performed because of the presence of cerebral oedema; no complications occurred, and the anti-edemigenic medical therapy was continued. The aim of the neurosurgical procedure was to avoid possible worsening of the patient’s condition, as well as compression on the brainstem. Twelve hours after the neurosurgical treatment, a brain CT scan showed a better definition of the ischaemic lesions, especially in the left cerebellar area, and improvement in the size of the ventricular system compared to that in the previous CT examination. 

**Figure 4:**
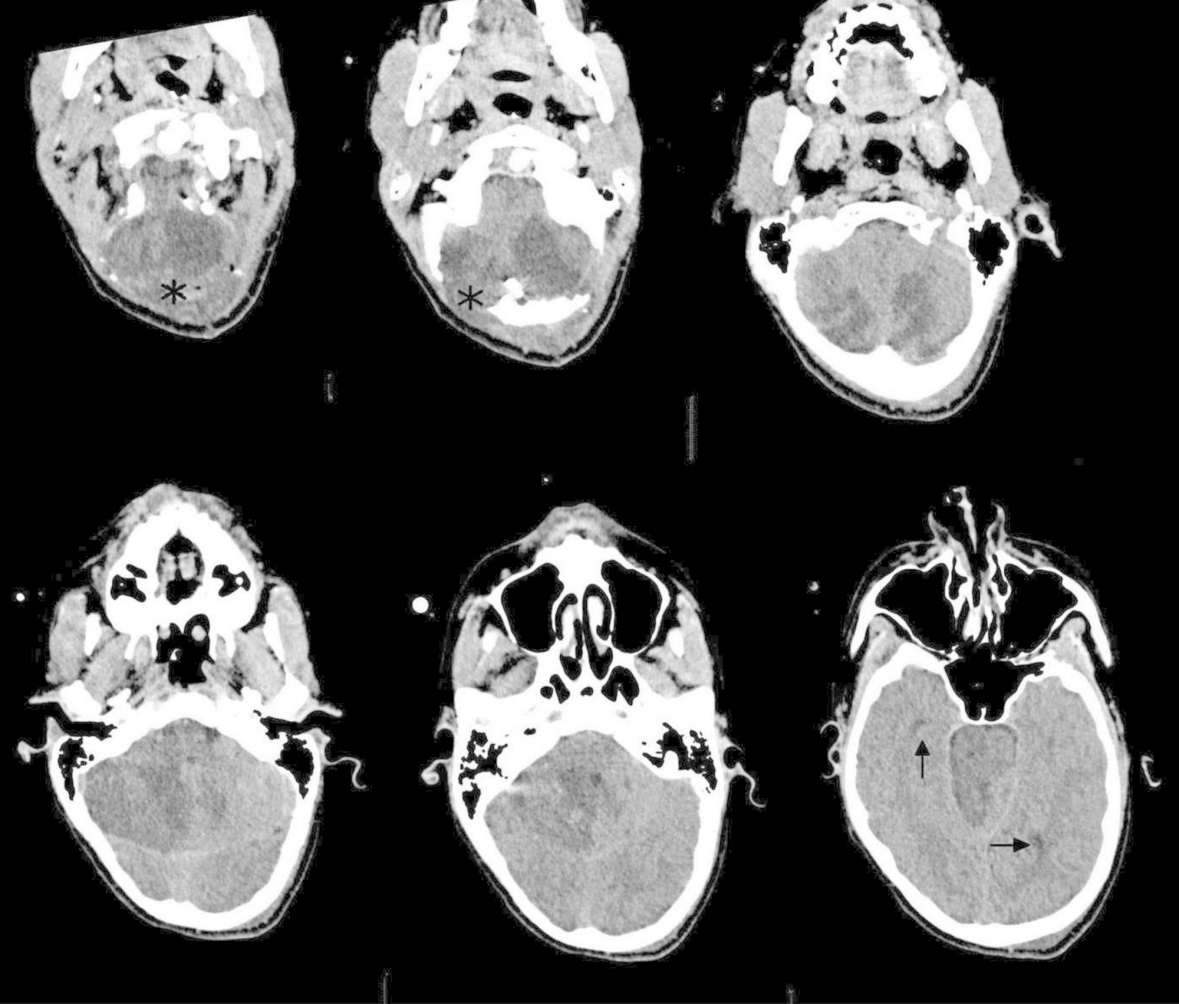
Computed tomography (CT) scan five days after neurosurgical treatment CT scan five days after occipital craniectomy (black stars) demonstrates physiological visualization of the cranial subarachnoid spaces due to the reduction of the compressive effect (black arrows).

Subsequently, the patient was evaluated five days after the endovascular treatment by both a brain CT scan and a clinical examination. The brain CT scan showed excellent visualization of the cranial subarachnoid spaces due to the reduction of the compressive effect (Figure 4a). The neurological examination revealed slight improvements in the conditions of the patient; he appeared alert, had hyposthenia in his left upper limb, and a bilateral deficiency in the sixth cranial nerve but there were no other neurological deficits.

In the following days, the patient remained stable, and one week after arriving at our hospital, he was transferred to another centre to continue his rehabilitation process. At the end of rehabilitation, the patient was functioning normally with a slight hyposthenia in his left upper limb.

## Discussion

The authors have decided to report this case mainly for three reasons. The first reason concerns the large time range between the onset of symptoms and endovascular treatment of acute ischaemic stroke. In our case, more than 72 hours had passed since the onset of symptoms and recanalization of the basilar apex.

With the development of the DAWN (DWI or CTP Assessment with Clinical Mismatch in the Triage of Wake-up and Late Presenting Strokes Undergoing Neurointervention With Trevo) and DEFUSE-3 (Endovascular Therapy Following Imaging Evaluation for Ischemic Stroke) clinical trials, the time for endovascular treatment in acute ischaemic stroke has been considerably extended [10-11]. Nevertheless, this consideration is not applicable to posterior fossa stroke in a paediatric patient. In any case, the time range considered was within 24 hours.

Currently, there is an expert consensus that the appropriate evaluation time for thrombectomy in cases of acute ischaemic stroke due to basilar artery occlusion is within six hours [12]. There is a lack of randomized clinical trials (RCTs) concerning these critical situations; however, there are two studies in the recruitment phase that are evaluating thrombectomy in posterior circulation within six [13] and 24 hours [14]. Considering recently published data on paediatric acute ischaemic stroke, the consideration of endovascular clot extraction techniques may be essential for improving recanalization rates and clinical outcomes [15].

In this specific case report, we reported the decision to perform an endovascular treatment. The decision was made in a multidisciplinary manner considering predominantly the young age of the patient and the favourable DWI pcASPECTs [8]. With the limitations related to a small number of cases, Hideaki et al. reported a favourable clinical outcome (modified Rankin Scale (mRS 0-2)) in 66.7% of patients with DWI pcASPECTs of a 6 or 7 [8]. The substantial integrity of the brainstem despite the presence of neurological symptoms, including irregularity of the pons (bilateral sixth nerve palsy), spinal cortical bundle (left hemiparesis), and ascending reticular activating system (drowsiness), were also fundamental in the decision to perform the endovascular procedure [16]. Recently, it was reported that mechanical thrombectomy was feasible and effective in patients with acute basilar artery occlusion, and the time to recanalization, age, and clinical condition at onset were significant independent predictors of a favourable outcome [17].

Gory et al. described a significantly higher rate of complete reperfusion with a shorter procedural time in patients with basilar artery occlusion by the ADAPT technique as the first-line strategy [18]. In this study, we performed the ADAPT technique with an AXS Catalyst™ 6F thromboaspiration catheter (Stryker Neurovascular, Fremont, CA, USA) with a fast and safe recanalization of the basilar artery, as well as a partial reconstruction of the dissected left vertebral artery.

The second interesting point that we want to emphasize is smart neurosurgical management. It has been well-reported that a preventive craniectomy for both anterior and posterior ischaemic stroke is of great importance in specific circumstances. Regarding decompressive hemicraniectomy in paediatric patients with a malignant middle cerebral artery infarction, the analysis of a few uncontrolled and retrospective case series suggested a difference between adults and children. The few studies available involving paediatric populations have shown a moderately favourable outcome (mRS 0-2) with the involvement of either hemisphere, a low Glasgow Coma Scale (GCS) score, the involvement of more than one vascular territory, and surgery beyond 48 hours of the stroke onset [19-20].

In our case, a preventive occipital craniectomy was promptly performed before the deterioration of the patient's general condition. In our mind, this point is crucial because this intervention once again preserved the brainstem.

The third and last point establishes that acute ischaemic stroke due to basilar artery occlusion is a life-threatening condition, and there is a need for acute ischaemic stroke to be managed in a high-volume hospital centre that is able to provide a multidisciplinary approach to the pathology [7, 12, 19].

## Conclusions

We believe that in the reported case, multidisciplinary management considerably increased the possibility of a favourable outcome in the patient. In an area in which uncertainties remain, a collaboration between specialists appears increasingly necessary.
